# Construction of gene causal regulatory networks using microarray data with the coefficient of intrinsic dependence

**DOI:** 10.1186/s40529-019-0268-8

**Published:** 2019-09-11

**Authors:** Li-yu Daisy Liu, Ya-Chun Hsiao, Hung-Chi Chen, Yun-Wei Yang, Men-Chi Chang

**Affiliations:** 1 Department of Agronomy, National Taiwan University, Taipei, 106 Taiwan; 2 Department of Horticulture and Landscape Architecture, National Taiwan University, Taipei, 106 Taiwan

**Keywords:** Gene regulatory network, Cause-effect relationship, Microarray, Coefficient of intrinsic dependence

## Abstract

**Background:**

In the past two decades, biologists have been able to identify the gene signatures associated with various phenotypes through the monitoring of gene expressions with high-throughput biotechnologies. These gene signatures have in turn been successfully applied to drug development, disease prevention, crop improvement, etc. However, ignoring the interactions among genes has weakened the predictive power of gene signatures in practical applications. Gene regulatory networks, in which genes are represented by nodes and the associations between genes are represented by edges, are typically constructed to analyze and visualize such gene interactions. More specifically, the present study sought to measure gene–gene associations by using the coefficient of intrinsic dependence (CID) to capture more nonlinear as well as cause-effect gene relationships.

**Results:**

A stepwise procedure using the CID along with the partial coefficient of intrinsic dependence (pCID) was demonstrated for the rebuilding of simulated networks and the well-known CBF-COR pathway under cold stress using Arabidopsis microarray data. The procedure was also applied to the construction of bHLH gene regulatory pathways under abiotic stresses using rice microarray data, in which OsbHLH104, a putative phytochrome-interacting factor (*OsPIF14*), and OsbHLH060, a positive regulator of iron homeostasis (*OsPRI1*) were inferred as the most affiliated genes. The inferred regulatory pathways were verified through literature reviews.

**Conclusions:**

The proposed method can efficiently decipher gene regulatory pathways and may assist in achieving higher predictive power in practical applications. The lack of any mention in the literature of some of the regulatory event may have been due to the high complexity of the regulatory systems in the plant transcription, a possibility which could potentially be confirmed in the near future given ongoing rapid developments in bio-technology.

## Background

Genes encode the information necessary for life, including the information determining an organism’s molecular biology and ability to translate proteins directly involved in different biological activities. Therefore, the quantity of mRNA transcripts, or the expression levels of mRNA, mainly represent the gene activities in a biological system at the molecular level (Le Novère 2015). Using high-throughput gene profiling technologies that have undergone rapid development over the past several decades, including microarray sequencing and next-generation sequencing, researchers are able to identify “gene signatures” defining genes whose expression levels are associated with particular traits or phenotypes under investigation (Ritchie et al. 2015). These gene signatures serve as biomarkers in a wide range of areas including drug development, disease diagnosis and prevention, and crop breeding, among others (Pérez-de-Castro et al. 2012; Gomez-Casati et al. 2013; Rykunov et al. 2016). Once such gene signatures have been recognized, questions such as “Do the gene signatures have synergistic interactions leading to the phenotypes?” and similar questions that presume gene–gene interactions that are well acknowledged in biosystems are commonly asked (Knight and Knight 2001; Segal et al. 2005). These questions can potentially be answered by simultaneously monitoring the expression levels of the regulators or regulatees using modern high-throughput gene expression technologies (Mantione et al. 2014; Liseron-Monfils and Ware 2015).

The Pearson correlation coefficient (PCC) is one of the mostly adopted methods for measuring the interactions among genes based on their expression levels (Song et al. 2012). Other measurements of association including the mutual information (MI) (Song et al. 2012), the partial Pearson correlation coefficient (pPCC) (de la Fuente et al. 2004), the coefficient of determination (CoD) (Higa et al. 2009), and the coefficient of intrinsic dependence (CID) (Liu et al. 2009) have also been used. The PCC and pPCC have the limitation of only identifying the linear relationship between any two gene expressions. In contrast, the CID requires neither distributional (e.g. normal) nor functional (e.g. linear) assumptions regarding gene expression data. CID(Y|X) designates the CID value of a variable Y given the information of another variable X. It takes any real value between 0 and + 1 inclusive. It is + 1 in the case of full dependence and is 0 in the case of independence. As the level of dependence increases, the CID value goes from 0 to 1. In past studies, the CID has been used in conjunction with the correlation coefficient to construct an estrogen receptor regulatory network (Liu et al. 2009), to infer and classify co-regulatory events using two transcription factors (Liu et al. 2012), and to perform gene set association analysis (GSAA) (Tsai and Liu 2013). We have further demonstrated that the CID outperforms conventional methods for the identification of different association patterns (Liu et al. 2009; Tsai and Liu 2013).

After potential regulator–regulatee interactions under particular conditions have been identified, those interactions can then be connected to one another if they share the same genes. Such interactions are considered to constitute the small units of entire gene regulatory networks (GRNs), which may be combined together to form more comprehensive networks that better represent the biosystems in question (Liseron-Monfils and Ware 2015). An inferred GRN can therefore provide insights into the relationships between the genes of interest for specific experiments and clarify the understanding of biological functions involving complex biological phenomena (Karlebach and Shamir 2008). More specifically, an inferred GRN consisting of nodes (which represent the genes) and edges (which represent significant gene–gene interactions) reflects the gene regulation events that may concurrently or sequentially occur under the conditions being investigated. Previous studies have revealed that the edges between nodes in a GRN are typically not randomly allocated but are presumably assigned according to the scale-free topological model (e.g., Liseron-Monfils and Ware 2015). This would result in a network in which most nodes, with the exception of a few highly connected ones, are connected by a sparse number of edges. In this study, we focused on the inference and reconstruction of GRNs using the results of microarray experiments. More ambitiously, we sought to model the causal relationships in a single GRN.

Within a GRN, the relationship between a transcription factor (TF) and its target genes is usually expected to consist of a causal relationship (Pilpel et al. 2001). A directed edge pointing from the TF to the target gene would be specified in order to emphasize the origin (source) and the consequence (target) in this kind of relationship. Compared to a co-expression GRN (i.e., a network with undirected edges), a cause-and-effect GRN requires in vitro or in silico evidence to assign the direction to an edge (Simcha et al. 2013). However, in vitro evidence may not be available at all times, while the symmetric property of some commonly used statistics limits the exploration of causal effects in a GRN (Hsing et al. 2005). In this study, we utilized the asymmetric property of the CID (i.e., CID(Y|X) is not necessarily equal to CID(X|Y)) to distinguish not only the associated gene pairs but the causes/effects in a gene regulation event. Asymmetry is a very unique feature of the CID, whereas some conventional methods, including the PCC, pPCC, and mutual information, provide symmetric results when considering the association between two variables. Other methods like the coefficient of determination may have limitations in terms of their capacity to be utilized on particular types of data (Liu 2005).

Another emphasis of this study was its utilization of a new measure derived from the CID to perform the stepwise selection of relevant genes for regulation path elongation. This new measure is called the partial coefficient of intrinsic dependence (pCID), a name which was inspired by the partial correlation coefficient (Hsiao and Liu 2016). The new measure was motivated by a difficulty encountered while using the multi-predictor CID described in a study by Liu et al. (2012) to identify relevant genes in the elongation step. Ideally, a proper stepwise procedure iteratively picks the relevant genes according to its magnitude of association to the target until no additional gene would significantly increase the amount of association. For example, CID(Source A|Target A1) would be significant while we also expect a significant CID(Source A|Target A1, Target A2) but an insignificant CID(Source A|Target A1, X) given an irrelevant gene X. However, due to the dominant effect of the most influential gene, i.e., Target A1, in the first step, CID(Source A|Target A1, X) would be mostly significant (Hsiao and Liu 2016). The pCID resolves this problem by decomposing only the information of the target variable which was not explained by the first predictor.

The present study further proposes a procedure to thoroughly reconstruct a GRN based on microarray gene expression data and using the CID along with the pCID. The procedure is first demonstrated on a simulated network. It is also applied to Arabidopsis microarray data to retrieve the CBF-COR pathway in Arabidopsis under cold stress in a “supervised” manner as well as to construct the rice bHLH gene regulatory network under abiotic stress in the seedling stage in an “unsupervised” manner. In the analysis of the CBF-COR pathway, it is known that cold-regulated genes (COR) are regulated by a family of transcription factors known as C-repeat binding factors (CBFs), including the transcription factors *CBF1*, *CBF2*, and *CBF3* (Fowler and Thomashow 2002; McKhann et al. 2008; Doherty et al. 2009). Experiments based on transgenic plants constitutively expressing *CBF1*, *CBF2*, and *CBF3* have suggested that the overexpression of the three genes induces the expression of similar gene sets, including *COR47*, *COR6.6*, and *COR78* (Gilmour et al. 2004). Relatedly, RNA blot analyses have been conducted to confirm that the overexpression of *CBF1* and *CBF3* would induce *COR15A*, *COR78*, *COR47*, and *COR6.6* gene expressions (Kasuga et al. 1999; Taji et al. 2002; Seki et al. 2002; Fowler and Thomashow 2002). McKhann et al. (2008) reported that the expression of *COR15B* may last for 5 weeks after cold treatment, while *COR47* is only expressed within 24 h after cold treatment. By constructing the CBF-COR pathway in the present study, we examined the sensitivity of the proposed procedure and gained more biological insights about the possible synergistic behaviors among three CBFs.

In the construction of the rice bHLH gene regulatory network, a larger family of bHLH (basic helix–loop–helix) transcription factors is of interest. The bHLH gene family in plants plays a principal role in developmental processes (Schaller 2012) that might govern the biotic and abiotic stress responses in plants (Fujita et al. 2006). However, the function of most rice bHLH genes remains unknown (Li et al. 2006). OsbHLH001 (*OsICE2*) and OsbHLH002 (*OsICE1*) are induced at the protein level in response to cold and salt stresses, but they are not affected by cold stress at the mRNA level (Nakamura et al. 2011). Previous studies have shown that OsbHLH006 (*RERJ1*) is up-regulated in response to wounding and drought stresses (Kiribuchi et al. 2005); the expression of OsbHLH009 (*OsMYC*), a homolog of *AtMYC2* in Arabidopsis, can be induced by drought stress (Baldoni et al. 2015); OsbHLH062 (OsbHLH1) could enhance cold tolerance (Li et al. 2006); OsbHLH148 is induced by salt stress and results in activation under cold stress (Seo et al. 2011); and OsbHLH152 (OsPILI1) could reduce internode elongation under drought stress (Todaka et al. 2012). In this study, we explored the responses of the OsbHLH genes and their potential target genes under abiotic stresses. We expect that the proposed procedure for reconstruct GRNs may be of assistance in reverse engineering biological pathways and better elucidating the understanding of bHLH gene regulatory processes.

## Methods

### Coefficient of intrinsic dependence (CID) and partial coefficient of intrinsic dependence (pCID)

The coefficient of intrinsic dependence, CID(Y|X), quantifies the statistical dependence between two genes (X, Y) observed from a sample of size *N* by assessing the discrepancies between the conditional distribution of Y, F(y|x), given the values of X and the marginal distribution of Y, F(y). The CID(Y|X) value can be estimated from the sample using the following equation:

$${\text{CID}}\left( {Y |X} \right) = \frac{1}{N} \times \frac{{\mathop \sum \nolimits_{i = 1}^{N} \mathop \sum \nolimits_{j = 1}^{N} \left[ {\hat{F}\left( {y_{i} |x_{j} } \right) - \hat{F}\left( {y_{i} } \right)} \right]^{2} }}{{\mathop \sum \nolimits_{i = 1}^{N} \hat{F}\left( {y_{i} } \right)\left[ {1 - \hat{F}\left( {y_{i} } \right)} \right]}}$$

where *x*_*i*_ and *y*_*i*_ are the observed value of *X* and *Y* in the *i*th object, respectively, and the distribution functions were estimated by nonparametric kernel smoothing method using the “np” package in R (version 0.40-13) (Hayfield and Racine 2008).

Inspired by the partial correlation coefficient, the partial coefficient of intrinsic dependence (pCID) further decomposes the variability of the distribution of the variable Y which was not explained by the conditional distribution of the variable Y given the first variable X_1_ but can be explained after adding a second variable X_2_ (Hsiao and Liu 2016). When the two distribution functions are not identical, the discrepancy between them implies the amount of partial dependence between *X*_2_ and *Y* given *X*_1_. Consequently, a recursive formula using CID values can be derived to compute the partial coefficient of intrinsic dependence of Y given X_2_ conditioned on X_1_:

$${\text{pCID}}\left( {Y |X_{2} ;X_{1} } \right) = \frac{{{\text{CID}}\left( {Y |X_{2} ,X_{1} } \right) - {\text{CID}}\left( {Y |X_{1} } \right)}}{{1 - {\text{CID}}\left( {Y |X_{1} } \right)}}.$$

The significance of the CID or pCID can then be assessed by the null distribution of the CID or pCID values by random permutations. That is, we randomly permuted the values of *Y* and re-computed the CID or pCID values. This was repeated 1000 times and yielded 1000 internal control values of the CID values under independence. The p value for each association relationship between two variables of interest was determined by the number of values greater than or equal to the estimated CID or pCID divided by 1001. Readers are referred to Hsiao and Liu (2016) for more mathematical details and toy examples for CID/pCID definitions as well as estimations.

### Strategy to construct the gene regulatory network

**Fig. 1:**
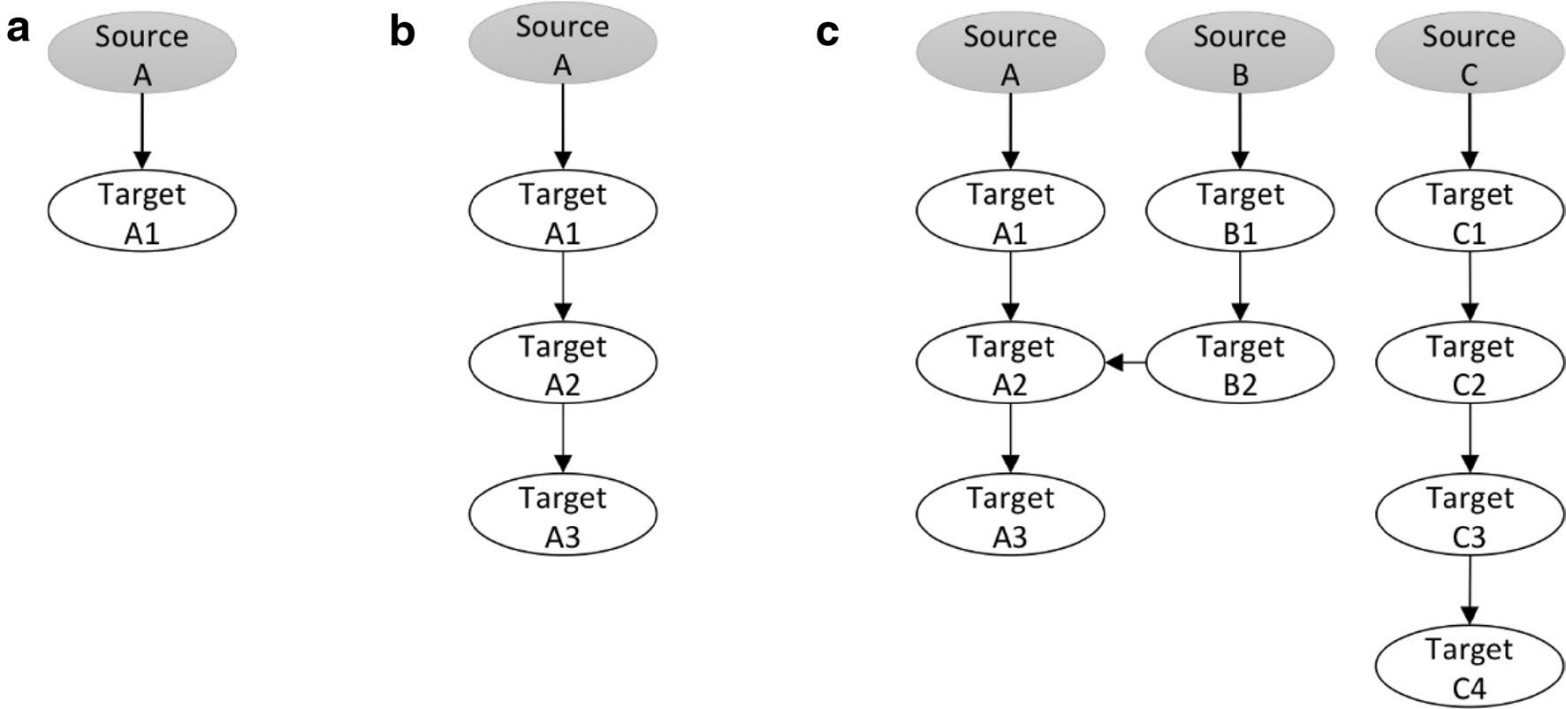
Diagram of gene regulatory network inference workflow. **a** Identification of a significantly associated gene pair. **b** Regulation path elongation. **c** Assembly of all identified regulation paths

The inference of a GRN has three steps (Fig. 1): (1) the identification of a significantly associated gene pair, (2) the regulation path elongation, and (3) the assembly of all the identified regulation paths. The basic principle of our GRN construction process designates gene Y as the source and gene X as the target, if CID(Y|X) > CID(X|Y). When prior knowledge about the preferable source genes is lacking, any gene in the collected data can possibly be the source as well as the target. Due to the dramatic amount of genes simultaneously monitored in a microarray experiment, we developed the following heuristic approach for the first two steps. Starting from a source gene *T*_0_, CID(*T*_0_|*T*_*i*_) is computed for one of the candidate target genes, *T*_*i*_, where

$${\text{CID}}\left( {T_{0} \left| {T_{i} } \right.} \right) = \frac{1}{N} \times \frac{{\sum\nolimits_{j = 1}^{N} {\sum\nolimits_{k = 1}^{N} {\left[ {\hat{F}\left( {T_{0} = t_{j} \left| {T_{i} = t_{k} } \right.} \right) - \hat{F}\left( {T_{0} = t_{j} } \right)} \right]^{2} } } }}{{\sum\nolimits_{j = 1}^{N} {\hat{F}\left( {T_{0} = t_{j} } \right)\left[ {1 - \hat{F}\left( {T_{0} = t_{j} } \right)} \right]} }}$$

$\hat{F}$’s are the corresponding distribution functions estimated from the sample using the nonparametric kernel smoothing method (Hsiao and Liu 2016), and *t*_*j*_ (and *t*_*k*_) are the *j*th (and *k*th) realization of gene *T*_0_ (and *T*_*j*_) (*j* or *k* = 1,…, *N*). In order to reduce the computation required of the programming, we occasionally eliminated some irrelevant candidate target genes which caused the CID(*T*_0_|*T*_*i*_) values to be insignificant (p-value > 0.05). Under these circumstances, the source gene *T*_0_ will be discarded as the origin of a regulation path when all CID(*T*_0_|*T*_*i*_) values are insignificant in the first run.

**Fig. 2:**
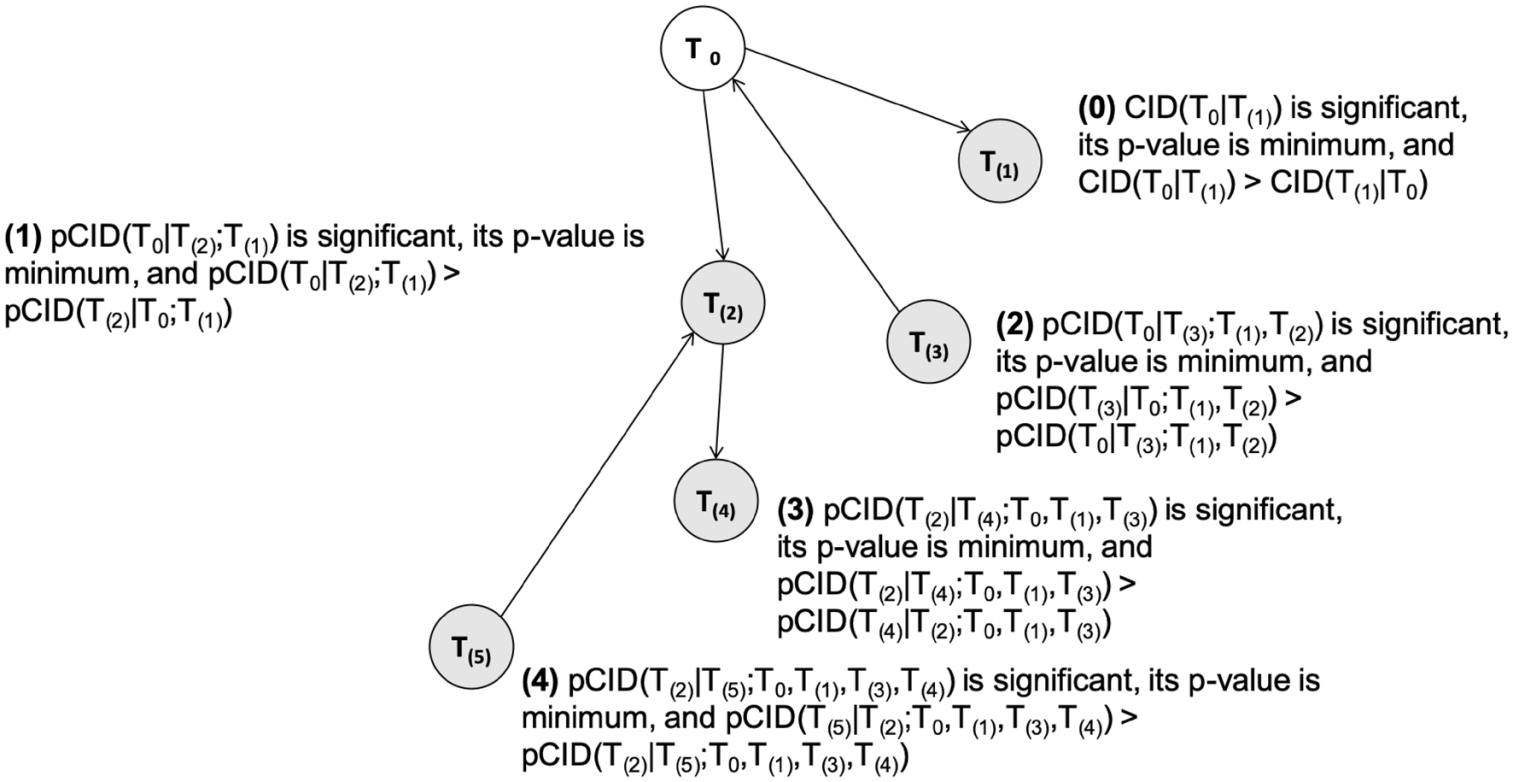
Illustration of the heuristic approach for regulation path elongation

In a set of *G* genes, we first specify the source gene *T*_0_. If CID(*T*_0_|*T*_(1)_) has the smallest p-value (or the largest CID value) among the results from all the candidate target genes, we connect the source gene *T*_*0*_ and the target *T*_(1)_. The direction is set from *T*_0_ to *T*_(1)_ if CID(*T*_0_|*T*_(1)_) is more significant than CID(*T*_(1)_|*T*_0_), or from *T*_(1)_ to *T*_0_, otherwise. The gene pair then proceeds to the elongation step. In the first step of elongation, pCID(*T*_0_|*T*_*j*_; *T*_(1)_) and pCID(*T*_(1)_| *T*_*j*_; *T*_0_) are computed for one of the remaining candidate genes, *T*_*j*_ (Fig. 2). If pCID(*T*_0_|*T*_(2)_; *T*_(1)_) is the most significant outcome (that is, the one with the smallest significant *p*-value) among the results from all the candidate target genes, we connect the genes *T*_0_ and *T*_(2)_, with the direction being from *T*_0_ to *T*_(2)_ if the *p*-value of pCID(*T*_0_|*T*_(2)_; *T*_(1)_) < the *p*-value of pCID(*T*_(2)_|*T*_0_;*T*_(1)_), or from *T*_(2)_ to *T*_0_, otherwise. Instead, if pCID(*T*_(1)_|*T*_(2)_; *T*_0_) is the most significant outcome (that is, the one with the smallest significant *p*-value) among the results from all the candidate target genes, we connect the genes *T*_(1)_ and *T*_(2)_, with the direction being from *T*_(1)_ to *T*_(2)_ if pCID(*T*_(1)_|*T*_(2)_; *T*_0_) is more significant than pCID(*T*_(2)_|*T*_(1)_;*T*_0_), or from *T*_(2)_ to *T*_(1)_, otherwise. This completes the first run of the elongation. In the *k*th run (*k* ≥ 2) of the elongation, all of the possible values of pCID(*S*|*T*_*j*_; {*T*_0_, *T*_(1)_, …, *T*_(*k*)_}\{*S*}) for *S* ∈ {*T*_0_, *T*_(1)_, …, *T*_(*k*)_} for the remaining (*G* − *k* − 1) genes are computed and result in (*k* + 1)(*G* − (*k* + 1)) pCID values. Suppose pCID(*S*|*T*_(*k* + 1)_; {*T*_0_, *T*_(1)_, …, *T*_(*k*)_}\{*S*}) is the most significant value, and we connect the node *S* and *T*_(k + 1)_; the direction is from *S* to *T*_(k + 1)_ if pCID(*S*|*T*_(*k* + 1)_; {*T*_0_, *T*_(1)_, …, *T*_(*k*)_}\{*S*}) is more significant than pCID(*T*_(*k* + 1)_|*S*; {*T*_0_, *T*_(1)_, …, *T*_(*k*)_}\{*S*}), or from *S* to *T*_(k + 1)_, otherwise. The elongation process continues until all of the pCID(*S*|*T*_*j*_; {*T*_0_, *T*_(1)_, …, *T*_(*e*)_}\{*S*}) values are insignificant (p-value > 0.05). The resulting network will contain *e* + 1 nodes (*T*_0_, *T*_(1)_, …, *T*_(*e*)_).

When the list of possible gene sources in the network is indicated by available biological evidence, the inference of the GRN can be simplified. In such cases, only pCID(*S*|*T*_*j*_; {*T*_0_, *T*_(1)_, …, *T*_(*k*)_}\{*S*}) for *S* ∈ {the possible gene sources} and their p-values are computed in the kth run (*k* ≥ 2) of the elongation. The whole elongation process then continues until all of the pCID(*S*|*T*_*j*_; {*T*_0_, *T*_(1)_, …, *T*_(*e*)_}\{*S*}) values are insignificant (p-value > 0.05) and result in a network with *e* + 1 nodes (*T*_0_, *T*_(1)_, …, *T*_(*e*)_).

### Simulation methods

**Fig. 3:**
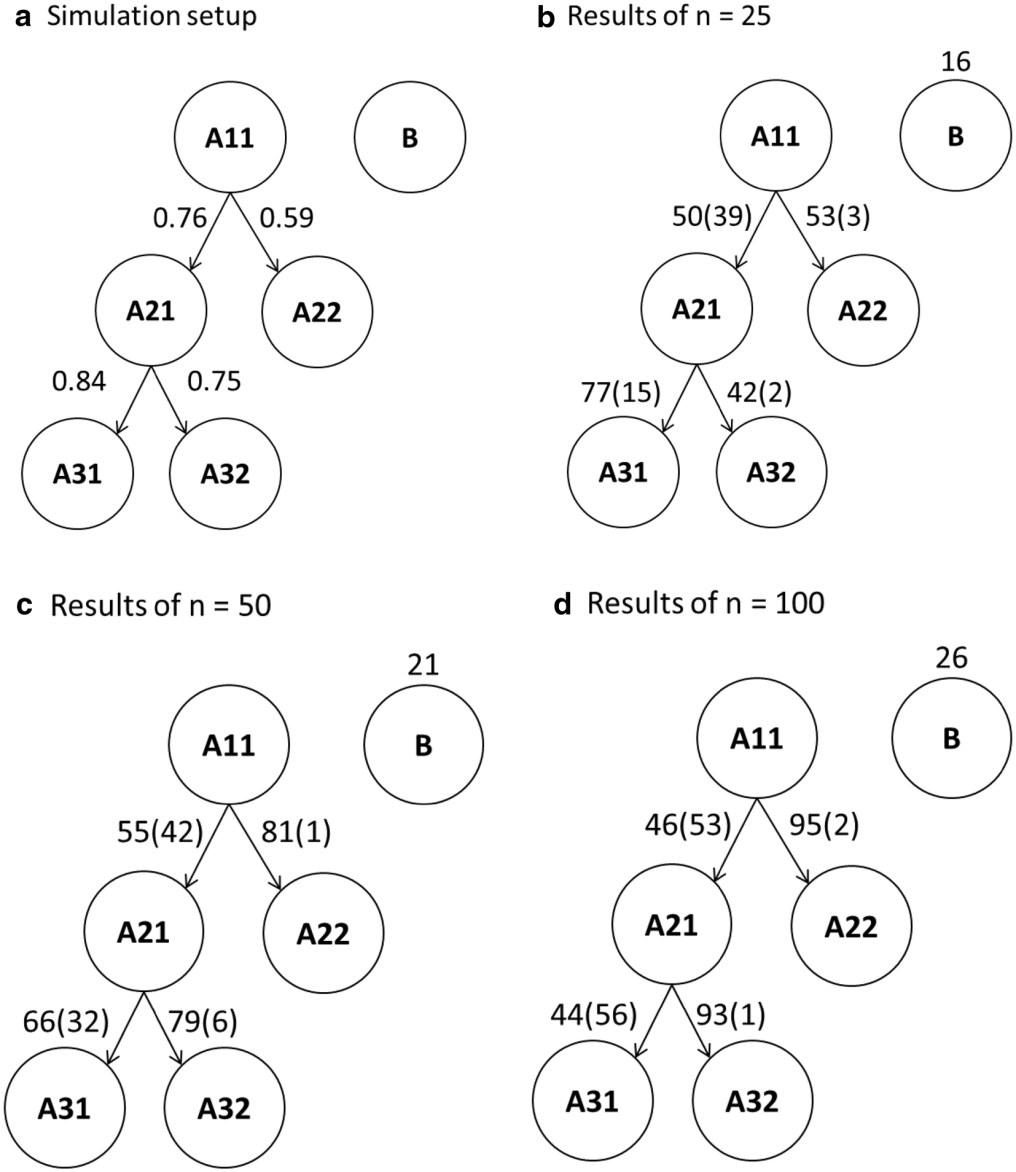
Reconstruction of the **a** simulated network using sample size, **b** n = 25, **c** n = 50, and **d** n = 100. The numbers next to the arrows in **a** are the probabilities of the expression levels of the target genes actually determined by the expression levels of the source genes. The numbers next to the arrows in **b**–**d** are the numbers of arrows pointing in correct directions (outside of the parentheses) and the numbers of arrows pointing in incorrect directions (in the parentheses)

The proposed procedure of GRN inference was examined in a simulation study. A pseudo network with six nodes (genes) was generated according to a normal mixture model (Fig. 3a). It contained one source node (A11), four target nodes (A21, A22, A31 and A32), and one node (B) independent of the others. The expression levels of nodes A11 and B were randomly generated from the normal distribution with means and standard deviations both equal to 1, *N*(1, 1). The expression levels of the target nodes were affected by two factors: the expression level and the binding efficiency of its direct source. This was intended to mimic the occasions in which (1) the transcription factor does not express so that the target gene is not regulated by the source gene, and (2) even if the source gene does express, the target gene may still not be regulated by the source gene due to the various binding efficiencies of the transcription factor. Let *S* and *T* denote the direct source and the target gene, respectively. In the simulated network, A11 was the direct source of {A21, A22} and A21 was the direct source of {A31, A32}. If the binding efficiency for this pair of *S* and *T* was set to be 100*b*%, then 100(1 − *b*)% of the objects in the sample would not be affected by the expression level of *S* and their expression levels would be generated from N(− 1, 1). The binding efficiency for {A11, A21}, {A11, A22}, {A21, A31}, and {A21, A32} were set to be 0.9, 0.7, 0.9, and 0.8, respectively (Table 1). For the 100*b*% objects for which the regulation did take place, if the expression level of *S* in the *i*th sample was *s*_*i*_, then the expression level of the *i*th sample was randomly generated from N(*s*_*i*_, 0.25) if *s*_*i*_ > 0 and from N(− 1, 0.25) if *s*_*i*_ < 0 (meaning that *S* was not expressed). The pseudo network was replicated 100 times with sample sizes of N = 25, 50, or 100.

**Table 1:** The binding efficiency (b) of the source gene (S) on the promoter region of the target gene (T), the probability that the expression level of the source gene is greater than 0, P(S > 0), and the probability that the expression levels of the target gene are actually determined by the expression levels of the source gene, P (S → T), in the simulated network with 6 nodes (Fig. 3a)

{*S*, *T*}	Binding efficiency (b)	P (*S* > 0)	P (*S* → *T*)
{A11, A21}	0.9	0.84	0.76
{A11, A22}	0.7	0.84	0.59
{A21, A31}	0.9	0.71	0.84
{A21, A32}	0.8	0.71	0.75

The approximate proportions of the gene expressions of the target gene actually determined by the expression levels of the source gene were expressed as P (*S* → *T*). Because the target gene can only be regulated by the source gene if the expression level of the source gene is greater than 0, we tabulated the two probabilities, P (*S* → *T*) and P(S > 0) denoting the probability that the expression level of the source gene is greater than 0, for all combinations of {*S*, *T*} in Table 1. In the simulated network, we deliberately set different efficiencies of regulation for each pair of {*S*, *T*} to examine the goodness of CID/pCID in detecting different levels of associations.

### Microarray expression data

The first dataset used was the expression data of *Arabidopsis thaliana* under cold stress to study the well-known CBF-COR pathway. This dataset can be downloaded from the Arabidopsis Information Resource (TAIR) database (Garcia-Hernandez et al. 2002). This data originally consisted of 22,810 probes and 52 samples (submission number ME00325). The tissues were treated in a 4 °C environment, and the expression levels were monitored after 0 (control), 0.5, 1, 3, 6, 12, or 24 h of treatment. The microarray expression raw dataset was first subjected to pre-processing using the RMA (Robust Multichip Average) method (Irizarry et al. 2003) and was log2 transformed. As an instance of supervised study, only probes related to CBF-COR regulation pathway in the microarray were collected for network construction according to their annotations.

A second dataset was used to study the bHLH pathway in rice (*Oryza sativa*). The expressions data can be downloaded from the NCBI-GEO database (Edgar et al. 2002) (accession numbers GSE6901 and GSE14275). The GSE6901 dataset includes the gene expressions of 7-day-old rice seedling samples under drought, salt, cold, and controlled conditions (three biological replicates of each condition). The GSE14275 dataset includes the gene expressions of 14-day-old rice seedling samples under heat and controlled conditions (three biological replicates of each condition). Expressed RNA samples were hybridized on Affymetrix microarrays (NCBI-GEO accession number GPL2025). The raw expression data of 51,279 probes from 18 samples were first subjected to pre-processing using the RMA method (Irizarry et al. 2003) and were log2 transformed. In this study, we were interested in the genes that were previously reported as related genes involved in the bHLH pathway (Li et al. 2006).

Some bHLH proteins recognize the G-box in the promoter region of their target genes (Gonzalez 2015). Among the bHLH-related genes, some of them can recognize and bind to the G-box according to Li et al. (2006). In this study, we also downloaded the gene sequences of the bHLH-related genes in the microarray from RAP-DB (version 7.0) (Sakai et al. 2013) to specify potential target genes containing G-box sequences in their promoter regions. The probes recognizing the bHLH-related genes and the probes containing G-box sequences were designated as the source and the candidate target genes, respectively, to construct the bHLH gene network. Note that there may have been some probes that served as both source and target genes since they not only could bind the G-box according to the literature but also had the G-box sequence in their promotor regions.

## Results

### Reconstruction of a pseudo network in the simulation

A pseudo network with six nodes (genes) was generated to assess the proposed procedure of GRN inference (Fig. 3a). Two source genes, A11 and B, were predetermined. The CID and pCID values as well as their p-values for a particular simulation of a sample with 25 realizations are shown in Table 2 as a demonstration of network reconstruction. Starting from A11, the results showed that CID(A11|A21) had the largest value (0.2146) and the smallest p-value (0.0010), such that A21 was selected as the first node connected to A11. Because CID(A11|A21) and CID(A21|A11) had the same significant *p*-value (0.0010) and because the CID(A11|A21) value (0.2146) was larger than the CID(A21|A11) value (0.1905), the direction was set from A11 to A21. The computation of pCID(A11|x; A21) and pCID(A21|x; A11) for another gene x followed and resulted in the selection of A22 as the second node connected to A11 due to the fact that pCID(A11|A22; A21) had the smallest p-value (0.0010) and the largest pCID value (0.1679). The direction was set from A11 to A22 because pCID(A11|A22; A21) had the same significant *p*-value (0.0010) as pCID(A22|A11; A21) but pCID(A11|A22; A21) = 0.1679 > pCID(A22|A11; A21) = 0.1460. Similarly, the third target and fourth target, A31 and A32, were selected based on pCID(A21|A31; A11, A22) and pCID(A21|A32; A11, A22, A31); both A31 and A32 were connected from A21 due to pCID(A21|A31; A11, A22) = 0.1751 > pCID(A31|A21; A11, A22) = 0.1707 (both had the same *p*-value) and [*p*-value of pCID(A21|A32; A11, A22, A31)] = 0.0070 < [*p*-value of pCID(A32|A11; A11, A22, A32)] = 0.8392. When considering the negative-control node B as the source node, it had all insignificant values of CID at the first step of GRN inference and was isolated from the other nodes. Therefore, the resulting network was identical to our setting shown in Fig. 3a.

**Table 2:** The estimated CID and pCID values in one simulation of sample size n = 25

CID/pCID	Estimated (p-value)	CID/pCID	Estimated (p-value)
CID(A11|A21)	*0.2146 (0.0010)*	CID(A21|A11)	0.1905 (0.0010)
CID(A11|A22)	0.2039 (0.0010)		
CID(A11|A31)	0.1019 (0.0060)		
CID(A11|A32)	0.0425 (0.0949)		
CID(A11|B)	0.0534 (0.0639)		
pCID(A11|A22; A21)	*0.1679 (0.0010)*	pCID(A22|A11; A21)	0.1460 (0.0030)
pCID(A11|A31; A21)	0.0288 (0.5075)		
pCID(A11|A32; A21)	0.0315 (0.4575)		
pCID(A11|B; A21)	0.0133 (0.6444)		
pCID(A21|A22; A11)	0.0027 (0.8462)		
pCID(A21|A31; A11)	0.1581 (0.0010)		
pCID(A21|A32; A11)	0.0934 (0.0120)		
pCID(A21|B; A11)	0.0363 (0.2957)		
pCID(A11|A31; A21, A22)	0.0285 (0.0889)		
pCID(A11|A32; A21, A22)	0.0129 (0.3247)		
pCID(A11|B; A21, A22)	− 0.0010 (0.6114)		
pCID(A21|A31; A11, A22)	*0.1751 (0.0020)*	pCID(A31|A21; A11, A22)	0.1707 (0.0020)
pCID(A21|A32; A11, A22)	0.1001 (0.0559)		
pCID(A21|B; A11, A22)	0.0523 (0.3187)		
pCID(A22|A31; A11, A21)	0.0007 (0.8961)		
pCID(A22|A32; A11, A21)	0.0059 (0.7872)		
pCID(A22|B; A11, A21)	− 0.0036 (0.9171)		
pCID(A11|A32; A21, A22, A31)	− 0.0019 (0.7772)		
pCID(A11|B; A21, A22, A31)	− 0.0055 (0.7972)		
pCID(A21|A32; A11, A22, A31)	*0.1165 (0.0070)*	pCID(A32|A21; A11, A22, A31)	0.0768 (0.8392)
pCID(A21|B; A11, A22, A31)	0.0272 (0.7772)		
pCID(A22|A32; A11, A21, A31)	0.0147 (0.7692)		
pCID(A22|B; A11, A21, A31)	0.0008 (0.9411)		
pCID(A31|A32; A11, A21, A22)	0.0523 (0.2567)		
pCID(A31|B; A11, A21, A22)	0.0196 (0.8192)		
pCID(A11|B; A21, A22, A31, A32)	0.0182 (0.3596)		
pCID(A21|B; A11, A22, A31, A32)	0.0257 (0.7453)		
pCID(A22|B; A11, A21, A31, A32)	0.0023 (0.9610)		
pCID(A31|B; A11, A21, A22, A32)	0.0156 (0.8611)		
pCID(A32|B; A11, A21, A22, A31)	0.0368 (0.7203)		
CID(B|A11)	0.0273 (0.6613)		
CID(B|A21)	0.0112 (0.6394)		
CID(B|A22)	0.0321 (0.2078)		
CID(B|A31)	0.0127 (0.5784)		
CID(B|A32)	0.0204 (0.3686)		

For simplicity, only the results of two pre-determined source genes, A11 and B, are shown

Italic signifies the combination having the largest CID/pCID value and the smallest p-value

We collected all the networks reconstructed in the simulations for N = 25, 50, and 100; networks consisting of the same set of nodes were grouped together and the groups that occurred at least 5 times are shown in Additional file 1: Figure S1. There were 14, 65, and 81 of 100 reconstructed networks that successfully recovered the correct network structure in simulations for N = 25, 50, and 100, respectively. Moreover, 54 and 10 of 100 reconstructed networks only correctly revealed the partial network for N = 25 and 50, respectively. A22 and A32 were discarded most often in the partial networks under the sample size N = 25 due to their lower proportions (59% and 0.76 × 0.75 = 57%, respectively) of gene expressions actually determined by the expression levels of A11 (Fig. 3a). Similarly, the edges A11–A22 and A21–A32 would be occasionally discarded under the sample size N = 50. The GRN could be mostly accurately reconstructed under the sample size N = 100.

In Fig. 3b–d, the numbers of all the connections between two nodes from 100 simulations under N = 25, 50 and, 100 are shown. When the sample size N = 25 and the source node was A11, there were 89% of networks that connected A11–A21 together, 92% of networks that connected A21–A31, 56% of networks that connected A11–A22, and 44% of networks that connected A21–A32, while 16% of the networks included the negative control node, B (Fig. 3b). When N = 50, 97%, 98%, 82%, and 85% of the networks contained the edges A11–A21, A21–A31, A11–A22, and A21–A32, respectively, while 21% of them had the negative control node, B (Fig. 3c). When N = 100, 99%, 100%, 97%, and 94% of the networks contained the edges A11–A21, A21–A31, A11–A22, and A21–A32, respectively, while 26% of them had the negative control node, B (Fig. 3d). When the negative control node, B, was set to be the source gene, 16% (Fig. 3b), 21% (Fig. 3c), and 26% (Fig. 3d) of the networks were significantly built at α = 0.05.

### Analysis of CBF-COR pathway under cold stress in *Arabidopsis thaliana*

**Fig. 4:**
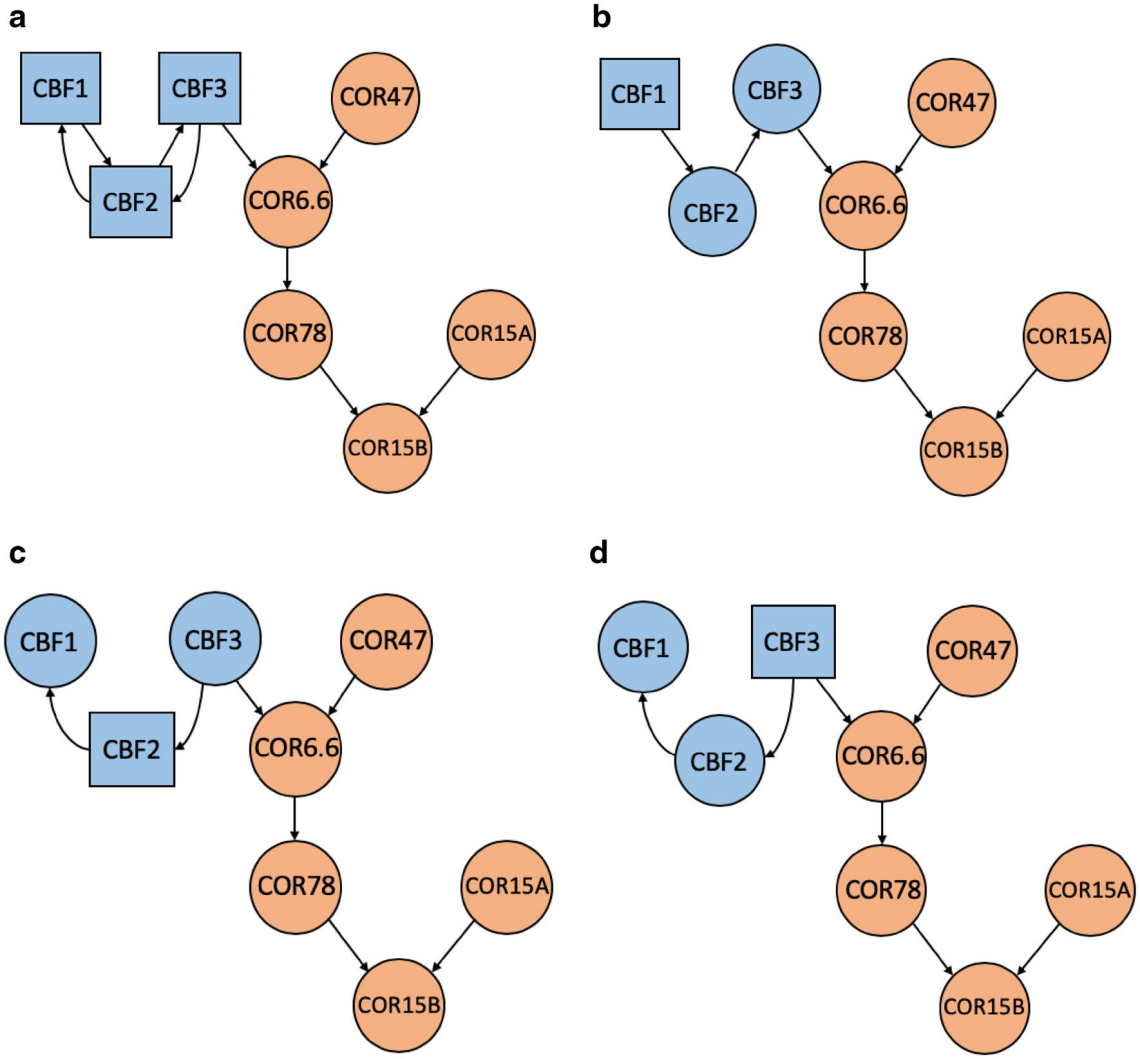
The reconstructed paths from the source CBF genes (blue rectangles) to the target CBF genes (blue circles) or target COR genes (orange circles), respectively, starting from **b** *CBF1*, **c** *CBF2*, and **d** *CBF3*. All the paths in **b**–**d** were combined to reconstruct the CBF-COR GRN in **a**

Using the microarray data of 44 samples, we intended to reconstruct the CBF-COR gene regulatory network (GRN) of eight genes related to cold stress in Arabidopsis. Three CBF TFs took turns being the source of the regulation path elongation, while the other probes were all considered as potential targets. Figure 4b–d present the reconstructed paths from the source CBF genes (blue rectangles) to the target CBF genes (blue circles) or the target COR genes (orange circles), respectively. The resulting paths starting from *CBF2* (Fig. 4c) and *CBF3* (Fig. 4d) were identical; the paths starting from *CBF1* (Fig. 4b) were similar to them except that the directions of the arrows between the CBF genes were opposite. We combined these paths to reconstruct the CBF-COR GRN shown in Fig. 4a. Both *CBF1* and *CBF3* were connected with *CBF2* in the GRN, while *CBF3* had direct contact with the studied downstream COR genes. The *COR6.6* was the first receiver of the information passed down from the CBF genes, which further influenced *COR78* and *COR15B*. In contrast, *COR47* and *COR15A* served as signal providers in the GRN.

### Construction of rice bHLH gene regulatory network under abiotic stress

**Fig. 5:**
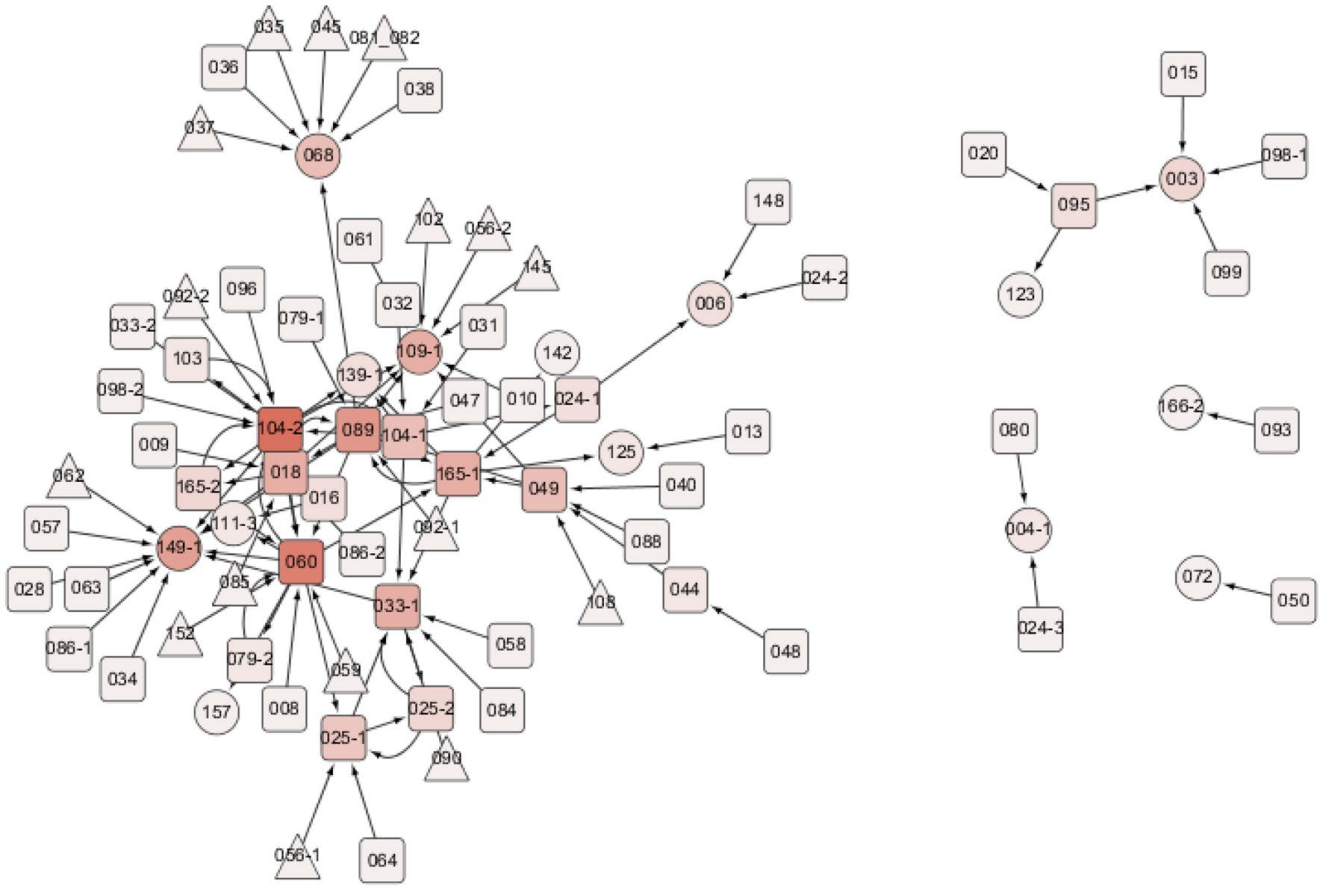
Triangle nodes indicate the bHLH probes capable of binding to G-box sequences (G-box binders) but not having G-box sequences in their promoter regions (being sources only); ellipse nodes indicate the bHLH probes having G-box sequences in their promoter regions but not known as the G-box binders (being targets only); round rectangle nodes are the G-box binders having G-box sequences in their promoter regions (being both sources and targets). The shade of the fill color in the node represents the total degree of the node. A list of all the probes (nodes) used in the study is provided in Additional file 2: Table S1, and the sub-network for each source probe is in Additional file 2: Table S2

The 61 known bHLH genes (72 probes) capable of binding to G-box sequences according to the literature (Gonzalez 2015) were assigned as source genes of the network and the 104 bHLH probes containing G-box sequences in their promoter regions were recruited as the potential targets. There were 54 probes that could be either sources or targets (Additional file 2: Table S1). All the sub-networks from all 72 source probes were assembled together to form the final version of the bHLH GRN in this study (Fig. 5).

We considered the source gene, OsbHLH104-1, to illustrate the elongation process in the bHLH GRN construction (Fig. 1b). Among those CID values of OsbHLH104-1 given to all 104 target probes, the largest one was CID(OsbHLH104-1| OsbHLH104-2) = 0.3926. Next, the largest significant pCID value was pCID(OsbHLH104-1|OsbHLH139-1; OsbHLH104-2) = 0.0738, provided that the OsbHLH104-1 was the source and OsbHLH104-2 was the first target, implying that OsbHLH139-1 was the second target in this particular sub-network. The elongation process stopped due to the fact that OsbHLH139-1 can be a target variable but not a source variable. When considering another source gene, OsbHLH056-1, the elongation process was stopped after adding one target gene, OsbHLH025-1, since all of the pCID(OsbHLH056-1|*T*_*j*_; OsbHLH025-1) values were insignificant (p-value > 0.05). All 72 source probes were processed using the same criteria (stopping when either encountering a target-only gene or having all insignificant CID/pCID values), and their resulting sub-networks are provided in Supp Table S2. Three of the 72 sources (OsbHLH083, OsbHLH144, and OsbHLH135) did not have significant CID values and their sub-networks were not further extended. Half of the 72 sub-networks expanded to only one target from the source; 28 sub-networks expanded to two targets; and 6 sub-networks expanded to three or four targets.

## Discussion

### The simulations verified the sensitivity and specificity of detecting directed gene–gene association by using CID/pCID

The medians and interquartile ranges of some CID and pCID values summarized from the 100 simulations are shown in Table 3. The CID values of A11 to a directed or undirected associated node were much larger than CID(A11|B)’s to the unassociated B. Also, it could be observed that, on average, CID(A11|A21) > CID(A11|A22), CID(A11|A31) > CID(A11|A32), and CID(A11|A21) > max(CID(A11|A31), CID(A11|A32)). The order of the average CID values followed the order of association strengths of the nodes to A11 (Fig. 3a). Therefore, a CID value can not only distinguish the existence of an association but also reflect the strength of the association and successfully pick the direct (or strongest) association among all possible connections. On the other hand, since 100% of the pCID(A21|A31; A11) values were significant at α = 0.05 and the medians of pCID(A21|A31; A11) were the largest in different sample sizes, A31 was the most likely to be selected after A21 eliminating the effects from A11. For N = 25, A22 was more likely selected after A31 and A21 [63% of the pCID(A11|A22; A21, A31) values were significant and pCID(A11|A22; A21, A31) had the largest median]. A32 [29% of the pCID(A21|A32; A11, A22, A31) values were significant and pCID(A21|A32; A11, A22, A31) had the largest median] might be selected as the last node associated with A11. With similar arguments, for N = 50, A21, A31, A32, and A22 were consecutively identified; for N = 100, A21, A31, A22, and A32 were consecutively identified.

**Table 3:** Summary of estimated CID and pCID values in 100 simulations

	n = 25		n = 50		n = 100	
	Median (IQR)	Sig. prop.	Median (IQR)	Sig. prop.	Median (IQR)	Sig. prop.
CID(A11|A21)	0.197 (0.053)	1.00	0.205 (0.053)	1.00	0.232 (0.038)	1.00
CID(A11|A22)	0.110 (0.057)	0.86	0.123 (0.052)	1.00	0.140 (0.033)	1.00
CID(A11|A31)	0.135 (0.063)	0.93	0.146 (0.061)	1.00	0.160 (0.035)	1.00
CID(A11|A32)	0.113 (0.071)	0.86	0.123 (0.050)	1.00	0.133 (0.038)	1.00
CID(A11|B)	0.028 (0.037)	0.06	0.016 (0.017)	0.13	0.012 (0.008)	0.16
CID(A21|A11)	0.194 (0.061)	1.00	0.202 (0.051)	1.00	0.231 (0.030)	1.00
pCID(A11|A22; A21)	0.078 (0.043)	0.74	0.082 (0.050)	0.96	0.084 (0.030)	1.00
pCID(A11|A31; A21)	0.036 (0.032)	0.22	0.030 (0.023)	0.55	0.017 (0.017)	0.83
pCID(A11|A32; A21)	0.031 (0.032)	0.19	0.022 (0.021)	0.40	0.012 (0.016)	0.72
pCID(A11|B; A21)	0.028 (0.035)	0.04	0.008 (0.019)	0.09	− 0.010 (0.015)	0.09
pCID(A21|A22; A11)	0.036 (0.031)	0.19	0.021 (0.022)	0.33	0.009 (0.014)	0.61
pCID(A22|A11; A21)	0.065 (0.045)	0.26	0.068 (0.036)	0.69	0.063 (0.025)	0.99
pCID(A21|A31; A11, A22)	0.119 (0.045)	0.96	0.124 (0.040)	1.00	0.128 (0.028)	1.00
pCID(A21|A32; A11, A22)	0.090 (0.047)	0.78	0.093 (0.044)	0.99	0.095 (0.024)	1.00
pCID(A21|B; A11, A22)	0.037 (0.032)	0.07	0.025 (0.019)	0.08	0.007 (0.017)	0.12
pCID(A11|A31; A21, A22)	0.034 (0.024)	0.12	0.026 (0.023)	0.33	0.017 (0.015)	0.68
pCID(A22|A31; A11, A21)	0.016 (0.020)	0.00	0.012 (0.014)	0.01	0.005 (0.012)	0.01
pCID(A31|A21; A11, A22)	0.111 (0.055)	0.77	0.112 (0.039)	0.99	0.125 (0.022)	1.00
pCID(A21|A32; A11, A22, A31)	0.048 (0.038)	0.29	0.056 (0.030)	0.88	0.058 (0.024)	1.00
pCID(A21|B; A11, A22, A31)	0.033 (0.029)	0.03	0.022 (0.015)	0.08	0.009 (0.019)	0.17
pCID(A11|A32; A21, A22, A31)	0.021 (0.021)	0.01	0.016 (0.016)	0.03	0.010 (0.010)	0.14
pCID(A22|A32; A11, A21, A31)	0.024 (0.021)	0.01	0.015 (0.020)	0.02	0.013 (0.013)	0.02
pCID(A31|A32; A11, A21, A22)	0.032 (0.026)	0.08	0.030 (0.021)	0.18	0.026 (0.015)	0.41
pCID(A32|A21; A11, A22, A31)	0.041 (0.037)	0.02	0.045 (0.022)	0.12	0.049 (0.017)	0.59
pCID(A11|B; A21, A22, A31, A32)	0.038 (0.036)	0.02	0.028 (0.022)	0.15	0.018 (0.018)	0.12
pCID(A21|B; A11, A22, A31, A32)	0.026 (0.023)	0.03	0.021 (0.016)	0.14	0.011 (0.014)	0.22
pCID(A22|B; A11, A21, A31, A32)	0.058 (0.030)	0.03	0.047 (0.023)	0.09	0.040 (0.018)	0.12
pCID(A31|B; A11, A21, A22, A32)	0.040 (0.026)	0.04	0.031 (0.022)	0.17	0.022 (0.017)	0.14
pCID(A32|B; A11, A21, A22, A31)	0.045 (0.032)	0.04	0.039 (0.022)	0.14	0.029 (0.020)	0.18
CID(B|A11)	0.027 (0.029)	0.08	0.017 (0.016)	0.07	0.012 (0.010)	0.10
CID(B|A21)	0.022 (0.023)	0.06	0.014 (0.013)	0.04	0.010 (0.007)	0.08
CID(B|A22)	0.019 (0.022)	0.03	0.011 (0.012)	0.05	0.008 (0.006)	0.04
CID(B|A31)	0.020 (0.024)	0.08	0.013 (0.015)	0.08	0.008 (0.009)	0.11
CID(B|A32)	0.019 (0.016)	0.05	0.013 (0.017)	0.11	0.008 (0.006)	0.09

*IQR* interquartile range, *sig. prop.* significant proportion

However, the false networks were built spontaneously without consensus. All of the false networks that started from B of the same combination of nodes only appeared less than or equal to five times in 100 simulations for N = 25, 50, and 100. Therefore, the CID/pCID method robustly identified the relationships between nodes and the extended the association network. The asymmetric property of the CID and pCID was utilized to infer causal effects in the network. When CID(Y|X) was more significant than CID(X|Y) or when pCID(Y|X; **Z**) was more significant than pCID(X|Y; **Z**), Y was claimed to be the source of the relationship between X and Y. In Fig. 3b–d, the numbers pointing in the correct directions are shown beside the arrows outside of the parentheses, whereas the numbers pointing in the incorrect directions are shown inside the parentheses. More than 90% of the significant A21–A32 and A11–A22 connections were with correct directions. Although the A11–A21 and A21–A31 associations were identified in more than 85% of the simulations for all the sample sizes, the percentages of arrows pointing in the correct directions might have been as few as 44% (A21–A31 for N = 100). The simulation results implied that a large sample size would aggravate the confusion regarding causality. For example, while 71 out of 86 (82.6%) arrows from A21 pointed to A31 for N = 25 and 67 out of 99 (67.7%) arrows from A21 pointed to A31 for N = 50, only 44 out of 100 (44.0%) arrows from A21 pointed to A31 for N = 100. We conjecture that the strong association between A11 and A21 would disguise the cause-effect relationship between them.

### Literature confirmed the results of CBF-COR pathway reconstruction in Arabidopsis

**Fig. 6:**
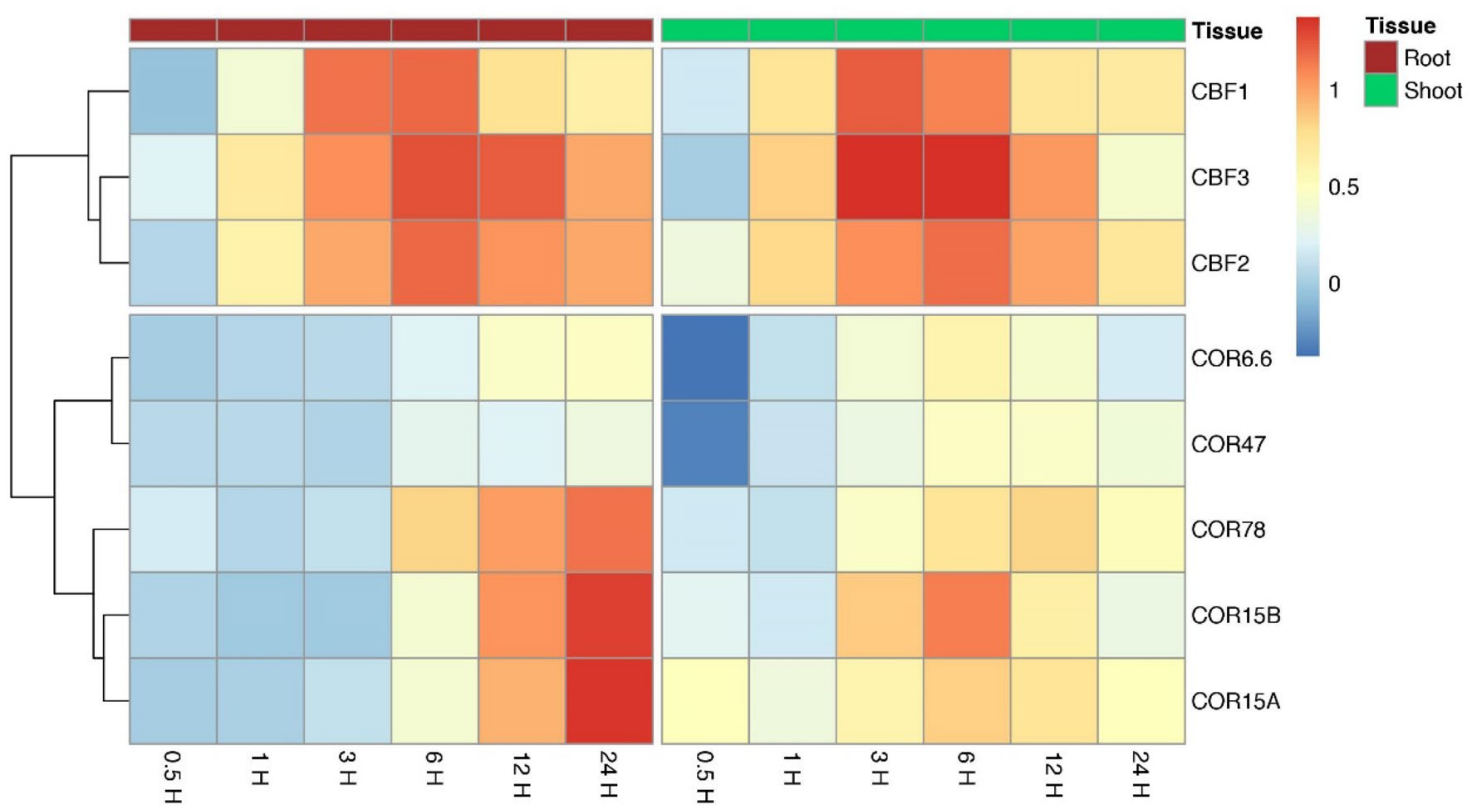
A heatmap and cluster analysis of the expression fold changes of CBF and COR genes at different time points after cold treatment relative to their corresponding control samples

C-repeat binding factors (CBF) bind to the promoter regions of downstream cold-regulated (COR) genes and induce COR genes expression under cold stress (Fowler and Thomashow 2002; McKhann et al. 2008; Doherty et al. 2009; Zhao et al. 2016). A heatmap and cluster analysis of the expression fold changes of CBF and COR genes at different time points after cold treatment relative to their corresponding control samples is shown in Fig. 6. The expressions of the CBF genes under cold stress increased earlier than those of the COR genes in both root and shoot tissues. Among them, *CBF3* had the highest relative expressions from 0.5 to 12 h(s) in root tissues and from 1 to 12 h(s) in shoot tissues; this was reflected in the outcome that *CBF3* was identified as the primary inducer of COR genes in our CID/pCID network results (Fig. 4a). In fact, it was evidenced that *COR47*, *COR78*, *COR15A*, *COR15B*, and *COR6.6* can be activated by *CBF3* under cold stress (Sakuma et al. 2006). The target genes, *COR47* and *COR6.6*, had similar expression levels, while *COR15A* and *COR15B* had similar expression levels. The CBF-COR GRN reconstructed by CID/pCID reflected their similarities by linking *COR47*-*COR6.6* and *COR15A*-*COR15B*. In particular, in root samples, the expressions of *COR78* were induced as early as 6H after cold treatment; it reacted before the other COR genes.

Experiments based on transgenic plants constitutively expressing *CBF1*, *CBF2*, and *CBF3* have suggested that overexpression of the three genes induces the expression of similar gene sets, including *COR47*, *COR6.6*, and *COR78* (Gilmour et al. 2004). RNA blot analysis has been conducted by others to confirm that the overexpression of *CBF1* and *CBF3* would induce *COR15A*, *COR78*, *COR47*, and *COR6.6* gene expressions (Kasuga et al. 1999; Taji et al. 2002; Seki et al. 2002; Fowler and Thomashow 2002). McKhann et al. (2008) reported that the expression of *COR15B* may last for 5 weeks after cold treatment, while *COR47* was only expressed within 24 h after cold treatment. The expression patterns of the microarray data investigated in this study were consistent with their findings (Fig. 6); *COR47* was set upstream relative to *COR15B* in the resulting network (Fig. 4a).

### Literature supported the discovered bHLH GRN in rice

A family of bHLH (basic helix–loop–helix) transcription factors in plants plays a principal role in various developmental processes (Ding et al. 2009; Chen and Chory 2011; Cui et al. 2016) that might be affected when plants suffer abiotic stresses. In this study, we explored the responses of the OsbHLH genes and their potential target genes under abiotic stresses. We combined all the resulting paths starting from all the sources to form the bHLH GRN (Fig. 5). The numbers in the node are the shortened ID numbers of the bHLH genes (for example, “001” stands for OsbHLH001 in rice). The resulting network involved 83 nodes and 107 edges, while some probes not connected to any other probe (nodes having “NA” for in-degree and out-degree in Additional file 2: Table S1) were excluded. The network obeys the power law of a biological network with an average degree of 2.58 (Additional file 1: Figure S2). The source having the largest out-degree value was OsbHLH104-2 (out-degree = 8), and the target having the largest in-degree value was OsbHLH149-1 (in-degree = 11). OsbHLH104-2 was also the most connected gene (in-degree + out-degree = 17), while OsbHLH060 was the second most connected one (in-degree + out-degree = 15).

According to a previous study, OsbHLH104 (LOC_Os07g05010), a putative phytochrome-interacting factor (OsPIF14), binds to N-boxes [CACG(A/C)G] in the OsDREB1B promoter and represses OsDREB1B gene expression, which reduces freezing tolerance in rice (Cordeiro et al. 2016). Interestingly, according to another study, PIF3/AtbHLH008 (At1g09530), an OsPIF14/OsbHLH104 homolog repressing photomorphogenesis, also has a negative impact on freezing tolerance by directly down-regulating the expression of CBFs in Arabidopsis (Jiang et al. 2017). These previous studies have indicated that OsbHLH104 plays a pivotal role in the connection between light and stress signaling.

According to one study, OsbHLH060 (LOC_Os08g04390), also known as *OsPRI1* [POSITIVE REGULATOR OF IRON HOMEOSTASIS 1], directly activates the expression of *OsIRO2* [*iron*-*related transcription factor 2/OsbHLH056* (LOC_Os01g72370)] and *OsIRO3* [*OsbHLH063* (LOC_Os03g26210)], which mediates rice responses to Fe deficiency (Zhang et al. 2017). Similarly, another study has indicated that the AtbHLH034 (At3g23210), 104 (At4g14410), 105/ILR3 [IAA-LEUCINE RESISTANT 3 (At5g54680)], and 115 (At1g51070) genes, which are highly homologous to the OsPRI1/OsbHLH060 gene, directly activate the transcription of the Ib subgroup *bHLH* genes, AtbHLH38/39/100/101 (At3g56970/At3g56980/At2g41240/At5g04150), and *PYE* [*POPEYE/AtbHLH047* (At3g47640), an *OsIRO3/OsbHLH063* homolog], that regulate Fe homeostasis in Arabidopsis (Wang et al. 2007; Long et al. 2010; Zhang et al. 2015; Li et al. 2016; Liang et al. 2017). Moreover, AtbHLH104 has been found to positively regulate several heavy metal detoxification-associated genes, such as *IREG2 (iron* *regulated 2)*, *MTP3* (*metal* *tolerance* *protein 3*), *HMA3* (*heavy* *metal* *ATPase 3*), and *NAS4* (*nicotianamine* *synthase 4*), which confer tolerance to cadmium stress in Arabidopsis (Yao et al. 2018). These previous studies suggest the possibility that OsbHLH060 is involved in cross-talk between Fe homeostasis and Cd stress tolerance.

### Utilization of CID/pCID on modern transcriptomic data

In this study, we demonstrated the construction of the GRN using microarray data. The main reason using microarray data is that after more than two decades of accumulation in the database, there are enough microarray expression samples for network construction. According to the simulation results, when the sample size is as small as 25 or 50, more than 50% of the resulting networks recovered only upstream regulatory events in the real network. Along with the advance of biotechnology, measuring global gene expression profiles by the whole transcriptome shotgun sequencing (RNA-seq) and single cell RNA-seq (scRNAseq) are common practice nowadays. It can be expect to have adequate sequencing data in the near future for network construction. It is worthwhile to mention that the gene expressions by sequencing are present by non-negative integers called ‘read counts’. The read counts are not normally distributed. Instead, the read counts are commonly analyzed as random samples from a Poisson or a negative binomial distribution (Robinson et al. 2010; Love et al. 2014, p. 2). The CID/pCID independent of distributional assumptions can be directly applied to sequencing data without a doubt. The non-distribution assumption of CID/pCID also implies the possibilities of applying CID/pCID on integrated transcriptomic, proteomic, metabolic, phenotypic data of different formats to construct bipartite or multipartite networks (Bass et al. 2013).

## Conclusion

Rapidly accumulated publicly accessible gene expression datasets have made it possible to systematically construct gene regulatory networks. In this study, we adopted a diverse dataset collected under different abiotic stresses. This strategy not only increased the sample size for statistical analysis but also made it possible to capture the gene–gene interactions under various circumstances simultaneously. Surely different combinations of gene expression datasets can be selected to better represent the population of interest based on the research purposes.

The proposed method makes use of the asymmetry of CID/pCID to determine the path directions in the gene regulatory network. The directions inferred in this study were then partly verified through literature reviews, although more finely designed experiments must be performed to piece together more solid biological evidence. In this study, we nonetheless demonstrated an exhaustive search in the simulation as well as heuristic methods in real datasets to accelerate the computation. The heuristic approach applied to the bHLH genes adopted as many resource/target bHLH genes as possible to demonstrate a mechanical way to build a comprehensive network. One can also pick fewer transcription factors or genes of interest in order to conduct an exhaustive search on a smaller scale.

In conclusion, this study proposed a three-step procedure to construct a directed gene regulatory network starting from the identification of incorporated genes connected as local pathways. The method is potentially applicable for deciphering causal events in proteomics, metabolomics, and epigenomics. Biologists can also customize the desired complexity of the inferred networks based on the complexity of the investigated biological systems. This flexible and constructive method may help to efficiently decipher gene regulatory pathways and achieve higher predictive power in practical applications.

## Supplementary information

**Additional file 1: Figure S1.** Summary of the networks reconstructed in the simulations for N = (A) 25, (B) 50, and (C) 100. Networks consisting of the same set of nodes were grouped together. Only groups occurred at least 5 times are shown. **Figure S2.** The scatter plot of the log(total degree) and the log(frequency) in the bHLH gene regulatory network. The inversely proportional trend between the log(total degree) and the log(frequency) indicates the resulting network obeys the power law.

**Additional file 2: Table S1.** The list of 122 bHLH-related probess in the microarray. The ones recognize the G-box in their targets’ promoter regions (Gonzalez 2015) are classified as the ‘Source’ genes; the ones containing G-box sequences in their promoter regions are classified as the ‘Target’ genes. ‘in.degree’ is the number of directed edge(s) using the probe as the target; ‘out.degree’ is the number of directed edge(s) using the probe as the source; ‘total.degree’ = ’in.degree’ + ’out.degree’. **Table S2.** The resulting sub-networks for bHLH source probes.

## Data Availability

The microarray experiments in this study can be downloaded from the Arabidopsis Information Resource (TAIR) database (https://www.arabidopsis.org/servlets/Search?type=expr&search_action=new_search) (submission number: ME00325), and the NCBI-GEO database (https://www.ncbi.nlm.nih.gov/geo/) (accession numbers: GSE6901 and GSE14275).

## Abbreviations

bHLH: basic helix–loop–helix
CBF: C-repeat binding factor
CID: the coefficient of intrinsic dependence
COR: cold-regulated gene
GSAA: gene set association analysis
GRN: gene regulatory network
NCBI-GEO: National Center for Biotechnology Information-Gene Expression Omnibus
PCC: Pearson correlation coefficient
pCID: partial coefficient of intrinsic dependence
pPCC: partial Pearson correlation coefficient
RAP-DB: Rice Annotation Project Database
RMA: robust multichip average
TAIR: the Arabidopsis Information Resource
TF: transcription factor

## References

[CR1] Baldoni E, Genga A, Cominelli E (2015). Plant MYB transcription factors: their role in drought response mechanisms. Int J Mol Sci.

[CR2] Bass JIF, Diallo A, Nelson J (2013). Using networks to measure similarity between genes: association index selection. Nat Methods.

[CR3] Chen M, Chory J (2011). Phytochrome signaling mechanisms and the control of plant development. Trends Cell Biol.

[CR4] Cordeiro AM, Figueiredo DD, Tepperman J (2016). Rice phytochrome-interacting factor protein OsPIF14 represses OsDREB1B gene expression through an extended N-box and interacts preferentially with the active form of phytochrome B. Biochim Biophys Acta BBA Gene Regul Mech.

[CR5] Cui J, You C, Zhu E (2016). Feedback regulation of DYT1 by interactions with downstream bHLH factors promotes DYT1 nuclear localization and anther development. Plant Cell.

[CR6] de la Fuente A, Bing N, Hoeschele I, Mendes P (2004). Discovery of meaningful associations in genomic data using partial correlation coefficients. Bioinformatics.

[CR7] Ding W, Yu Z, Tong Y (2009). A transcription factor with a bHLH domain regulates root hair development in rice. Cell Res.

[CR8] Doherty CJ, Van Buskirk HA, Myers SJ, Thomashow MF (2009). Roles for Arabidopsis CAMTA transcription factors in cold-regulated gene expression and freezing tolerance. Plant Cell.

[CR9] Edgar R, Domrachev M, Lash AE (2002). Gene Expression Omnibus: NCBI gene expression and hybridization array data repository. Nucleic Acids Res.

[CR10] Fowler S, Thomashow MF (2002). Arabidopsis transcriptome profiling indicates that multiple regulatory pathways are activated during cold acclimation in addition to the CBF cold response pathway. Plant Cell.

[CR11] Fujita M, Fujita Y, Noutoshi Y (2006). Crosstalk between abiotic and biotic stress responses: a current view from the points of convergence in the stress signaling networks. Curr Opin Plant Biol.

[CR12] Garcia-Hernandez M, Berardini T, Chen G (2002). TAIR: a resource for integrated Arabidopsis data. Funct Integr Genomics.

[CR13] Gilmour SJ, Fowler SG, Thomashow MF (2004). Arabidopsis transcriptional activators CBF1, CBF2, and CBF3 have matching functional activities. Plant Mol Biol.

[CR14] Gomez-Casati DF, Zanor MI, Busi MV (2013). Metabolomics in plants and humans: applications in the prevention and diagnosis of diseases. Biomed Res Int.

[CR15] Gonzalez DH (2015). Plant transcription factors: evolutionary, structural and functional aspects.

[CR01] Hayfield T, Racine JS (2008). Nonparametric econometrics: the np package. J Stat Softw.

[CR16] Higa CHA, Hashimoto RF, Hirata R, et al (2009) Inference of gene regulatory network using temporal coefficient of determination obtained from ergodic Markov chains. In: 2009 IEEE International Workshop on Genomic Signal Processing and Statistics. pp 1–4

[CR17] Hsiao Y-C, Liu L-YD (2016). A stepwise approach of finding dependent variables via coefficient of intrinsic dependence. J Comput Biol.

[CR18] Hsing T, Liu L-Y, Brun M, Dougherty ER (2005). The coefficient of intrinsic dependence (feature selection using el CID). Pattern Recognit.

[CR19] Irizarry RA, Hobbs B, Collin F (2003). Exploration, normalization, and summaries of high density oligonucleotide array probe level data. Biostatistics.

[CR20] Jiang B, Shi Y, Zhang X (2017). PIF3 is a negative regulator of the CBF pathway and freezing tolerance in Arabidopsis. Proc Natl Acad Sci.

[CR21] Karlebach G, Shamir R (2008). Modelling and analysis of gene regulatory networks. Nat Rev Mol Cell Biol.

[CR22] Kasuga M, Liu Q, Miura S (1999). Improving plant drought, salt, and freezing tolerance by gene transfer of a single stress-inducible transcription factor. Nat Biotechnol.

[CR23] Kiribuchi K, Jikumaru Y, Kaku H (2005). Involvement of the basic helix-loop-helix transcription factor RERJ1 in wounding and drought stress responses in rice plants. Biosci Biotechnol Biochem.

[CR24] Knight H, Knight MR (2001). Abiotic stress signalling pathways: specificity and cross-talk. Trends Plant Sci.

[CR25] Le Novère N (2015). Quantitative and logic modelling of molecular and gene networks. Nat Rev Genet.

[CR26] Li X, Duan X, Jiang H (2006). Genome-wide analysis of basic/helix-loop-helix transcription factor family in rice and Arabidopsis. Plant Physiol.

[CR27] Li X, Zhang H, Ai Q (2016). Two bHLH transcription factors, bHLH34 and bHLH104, regulate iron homeostasis in *Arabidopsis thaliana*. Plant Physiol.

[CR28] Liang G, Zhang H, Li X (2017). bHLH transcription factor bHLH115 regulates iron homeostasis in *Arabidopsis thaliana*. J Exp Bot.

[CR29] Liseron-Monfils C, Ware D (2015). Revealing gene regulation and associations through biological networks. Curr Plant Biol.

[CR30] Liu LD (2005). Coefficient of intrinsic dependence: a new measure of association.

[CR31] Liu L-YD, Chen C-Y, Chen M-JM (2009). Statistical identification of gene association by CID in application of constructing ER regulatory network. BMC Bioinform.

[CR32] Liu L-YD, Chang L-Y, Kuo W-H (2012). In silico prediction for regulation of transcription factors on their shared target genes indicates relevant clinical implications in a breast cancer population. Cancer Inform.

[CR33] Long TA, Tsukagoshi H, Busch W (2010). The bHLH transcription factor POPEYE regulates response to iron deficiency in Arabidopsis roots. Plant Cell.

[CR34] Love MI, Huber W, Anders S (2014). Moderated estimation of fold change and dispersion for RNA-seq data with DESeq2. Genome Biol.

[CR35] Mantione KJ, Kream RM, Kuzelova H (2014). Comparing bioinformatic gene expression profiling methods: microarray and RNA-seq. Med Sci Monit Basic Res.

[CR36] McKhann HI, Gery C, Bérard A (2008). Natural variation in CBF gene sequence, gene expression and freezing tolerance in the Versailles core collection of *Arabidopsis thaliana*. BMC Plant Biol.

[CR37] Nakamura J, Yuasa T, Huong TT (2011). Rice homologs of inducer of *CBF* expression (OsICE) are involved in cold acclimation. Plant Biotechnol.

[CR38] Pérez-de-Castro AM, Vilanova S, Cañizares J (2012). Application of genomic tools in plant breeding. Curr Genomics.

[CR39] Pilpel Y, Sudarsanam P, Church GM (2001). Identifying regulatory networks by combinatorial analysis of promoter elements. Nat Genet.

[CR40] Ritchie ME, Phipson B, Wu D (2015). limma powers differential expression analyses for RNA-sequencing and microarray studies. Nucleic Acids Res.

[CR41] Robinson MD, McCarthy DJ, Smyth GK (2010). edgeR: a bioconductor package for differential expression analysis of digital gene expression data. Bioinformatics.

[CR42] Rykunov D, Beckmann ND, Li H (2016). A new molecular signature method for prediction of driver cancer pathways from transcriptional data. Nucleic Acids Res.

[CR43] Sakai H, Lee SS, Tanaka T (2013). Rice Annotation Project Database (RAP-DB): an integrative and interactive database for rice genomics. Plant Cell Physiol.

[CR44] Sakuma Y, Maruyama K, Osakabe Y (2006). Functional analysis of an Arabidopsis transcription factor, DREB2A, involved in drought-responsive gene expression. Plant Cell.

[CR45] Schaller GE (2012). Ethylene and the regulation of plant development. BMC Biol.

[CR46] Segal E, Friedman N, Kaminski N (2005). From signatures to models: understanding cancer using microarrays. Nat Genet.

[CR47] Seki M, Narusaka M, Kamiya A (2002). Functional annotation of a full-length Arabidopsis cDNA collection. Science.

[CR48] Seo J-S, Joo J, Kim M-J (2011). OsbHLH148, a basic helix-loop-helix protein, interacts with OsJAZ proteins in a jasmonate signaling pathway leading to drought tolerance in rice. Plant J.

[CR49] Simcha DM, Younes L, Aryee MJ, Geman D (2013). Identification of direction in gene networks from expression and methylation. BMC Syst Biol.

[CR50] Song L, Langfelder P, Horvath S (2012). Comparison of co-expression measures: mutual information, correlation, and model based indices. BMC Bioinform.

[CR51] Taji T, Ohsumi C, Iuchi S (2002). Important roles of drought- and cold-inducible genes for galactinol synthase in stress tolerance in *Arabidopsis thaliana*. Plant J Cell Mol Biol.

[CR52] Todaka D, Nakashima K, Shinozaki K, Yamaguchi-Shinozaki K (2012). Toward understanding transcriptional regulatory networks in abiotic stress responses and tolerance in rice. Rice.

[CR53] Tsai C-A, Liu L-YD (2013). Identifying gene set association enrichment using the coefficient of intrinsic dependence. PLoS ONE.

[CR54] Wang H-Y, Klatte M, Jakoby M (2007). Iron deficiency-mediated stress regulation of four subgroup Ib BHLH genes in *Arabidopsis thaliana*. Planta.

[CR55] Yao X, Cai Y, Yu D, Liang G (2018). bHLH104 confers tolerance to cadmium stress in *Arabidopsis thaliana*. J Integr Plant Biol.

[CR56] Zhang J, Liu B, Li M (2015). The bHLH transcription factor bHLH104 interacts with IAA-LEUCINE RESISTANT3 and modulates iron homeostasis in Arabidopsis. Plant Cell.

[CR57] Zhang H, Li Y, Yao X (2017). Positive regulator of iron homeostasis1, OsPRI1, facilitates iron homeostasis. Plant Physiol.

[CR58] Zhao C, Zhang Z, Xie S (2016). Mutational evidence for the critical role of CBF transcription factors in cold acclimation in Arabidopsis. Plant Physiol.

