# Supplementary material for: Construction of gene causal regulatory networks using microarray data with the coefficient of intrinsic dependence
Source: Bot Stud. 2019 Sep 11;60:22. doi: 10.1186/s40529-019-0268-8 (PMC6738364; doi:10.1186/s40529-019-0268-8)
Supplement: Supplementary file 1 — Additional file 1: Figure S1. Summary of the networks reconstructed in the simulations for N = (A) 25, (B) 50, and (C) 100. Networks consisting of the same set of nodes were grouped together. Only groups occurred at least 5 times are shown. Figure S2. The scatter plot of the log(total degree) and the log(frequency) in the bHLH gene regulatory network. The inversely proportional trend between the log(total degree) and the log(frequency) indicates the resulting network obeys the power law. [file 40529_2019_268_MOESM1_ESM.docx]

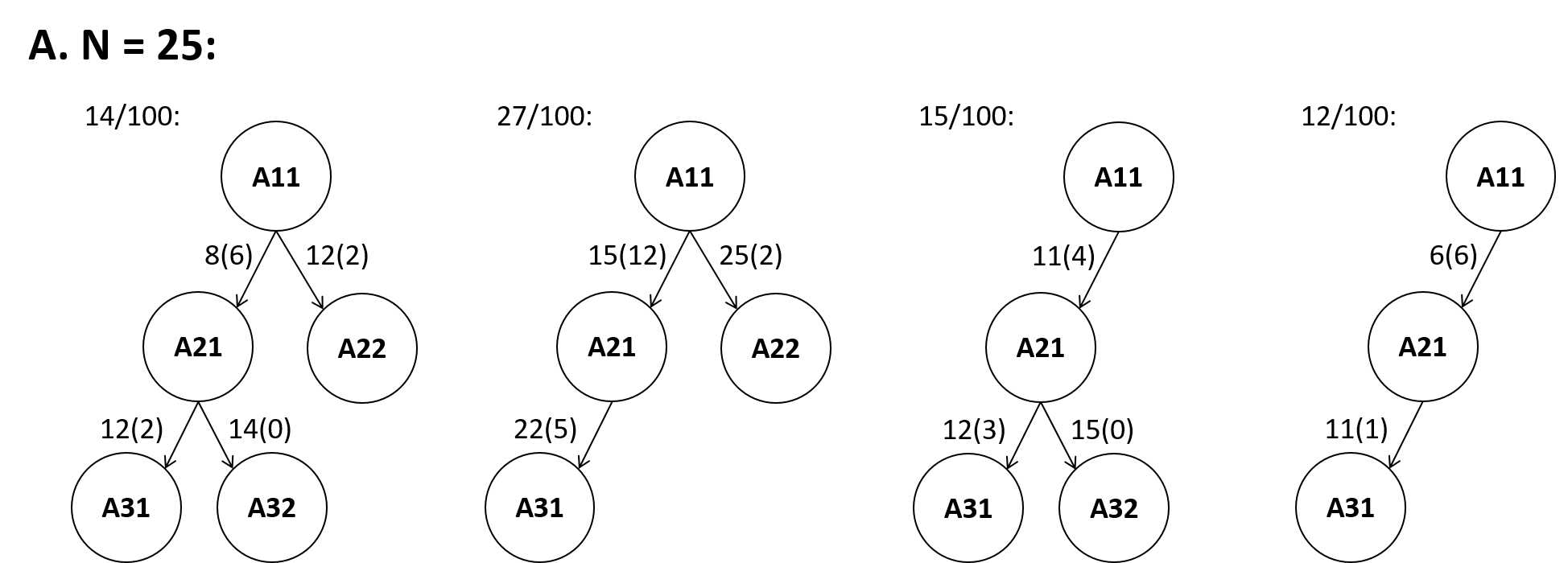


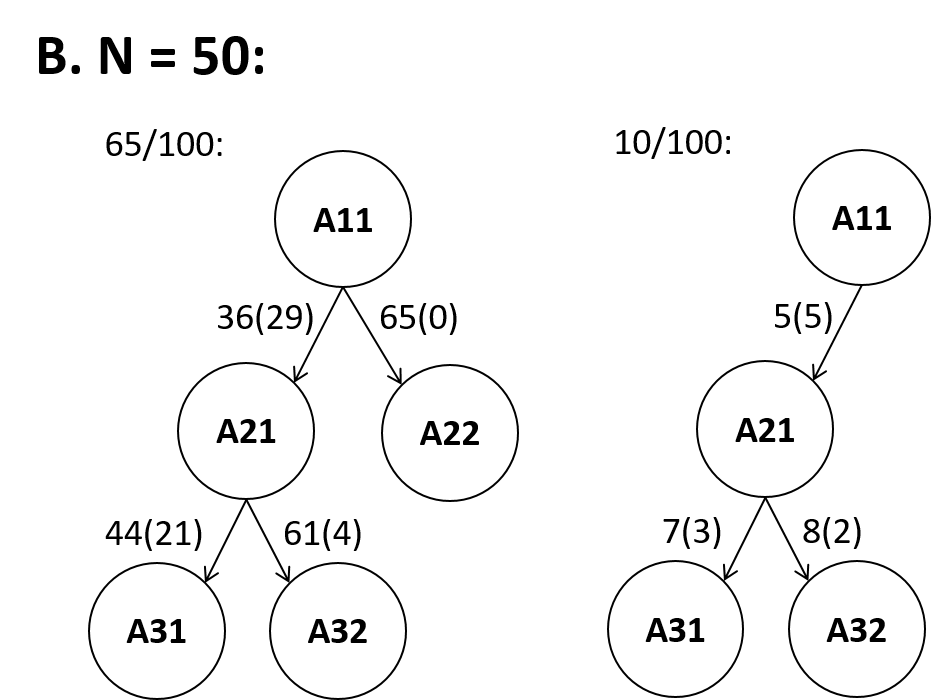

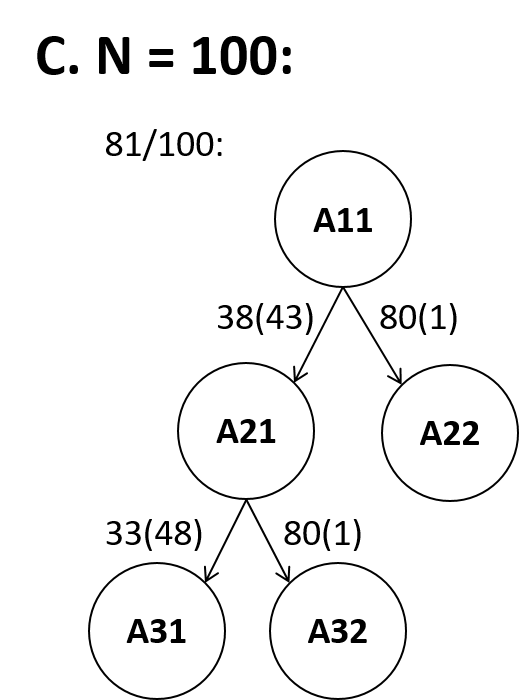


**Figure S1.** Summary of the networks reconstructed in the simulations for N = (A) 25, (B) 50, and (C) 100. Networks consisting of the same set of nodes were grouped together. Only groups occurred at least 5 times are shown.


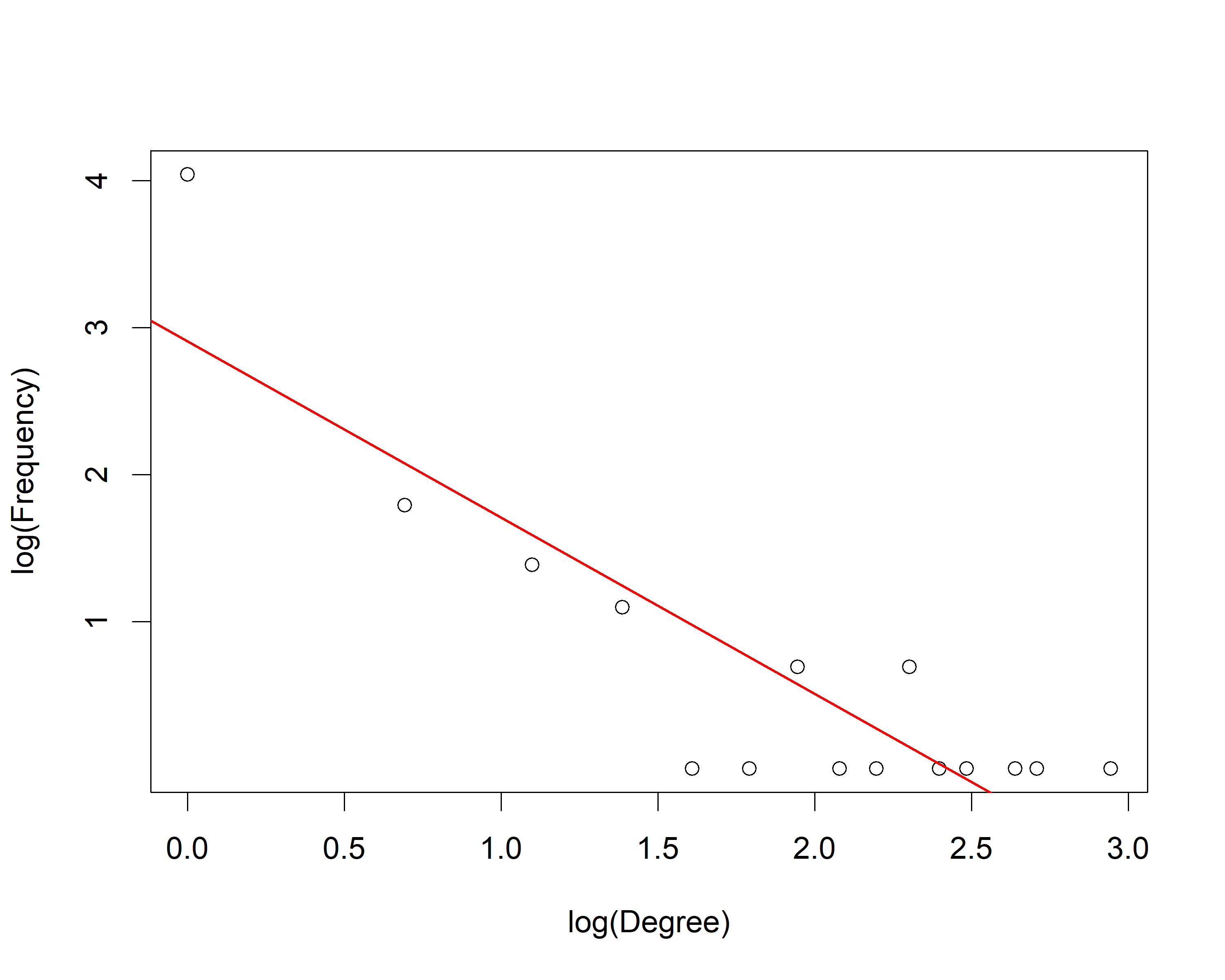


**Figure S2.** The scatter plot of the log(total degree) and the log(frequency) in the bHLH gene regulatory network. The inversely proportional trend between the log(total degree) and the log(frequency) indicates the resulting network obeys the power law.
